# Clinical and Molecular Features of Patients With Congenital Disorders of Glycosylation in Japan

**DOI:** 10.1002/jmd2.70011

**Published:** 2025-04-04

**Authors:** Nobuhiko Okamoto, Machiko Kadoya, Yoshinao Wada

**Affiliations:** ^1^ Department of Medical Genetics Osaka Women's and Children's Hospital Izumi Japan; ^2^ Department of Molecular Medicine Research Institute, Osaka Women's and Children's Hospital Izumi Japan; ^3^ Department of Obstetric Medicine Osaka Women's and Children's Hospital Izumi Japan

**Keywords:** ATP6V0A2‐CDG, NGLY1‐CDDG, PMM2‐CDG, SLC35A2‐CDG

## Abstract

Congenital disorders of glycosylation (CDG) are a heterogeneous group of diseases caused by defects in various steps of the glycosylation pathway. There are over 200 known human glycosylation‐related disorders. Many of these defects lead to multisystemic manifestations, commonly involving the central nervous system, with symptoms ranging from mild to severe. The phenotypic presentation of CDG can vary significantly. Identifying altered protein glycosylation is crucial for accurate diagnosis. Our research institute has contributed to the CDG diagnostic support center in Japan, developing new analytical techniques utilizing mass spectrometry. These techniques allow for the identification of defects in *N*‐glycosylation, *O*‐glycosylation, and combined glycosylation pathways. Advances in genetic analysis, including whole exome sequencing, have revealed that certain types of CDG are more prevalent than previously recognized. We have contributed to the molecular diagnosis of 66 CDG patients in Japan. Although PMM2‐CDG is the most common form of CDG, it was only detected in 17% of the patients in the present study, suggesting that its incidence is much lower in Japan compared to European countries. We also conducted a comprehensive review of case reports of CDG in Japan, further describing the clinical and molecular spectrum of the disease in this population.

1

**Summary**

- Congenital disorders of glycosylation (CDG) are a diverse group of diseases caused by glycosylation pathway defects, often affecting the central nervous system with varying severity.
- Our research institute has supported CDG diagnosis in Japan by developing mass spectrometry techniques to analyze N‐glycosylation, O‐glycosylation, and combined defects.
- Genetic advances, including whole exome sequencing, have identified previously underrecognized CDG types.
- We diagnosed 66 CDG patients in Japan, with PMM2‐CDG found in only 17%, suggesting a lower incidence than in Europe. Additionally, we reviewed Japanese CDG cases to better define their clinical and molecular characteristics.

## 2 Introduction

Congenital disorders of glycosylation (CDG) are heterogeneous diseases caused by defects in various steps of the glycosylation pathway. Jaeken et al. [1] described Belgian identical twin sisters with a disorder characterized by psychomotor retardation suggestive of a demyelinating disease and multiple serum glycoprotein abnormalities. The number of newly described CDG has increased exponentially, with currently 189 genes associated with 200 disorder phenotypes [2]. Many of these defects lead to multisystemic manifestations, which commonly involve the central nervous system with mild‐to‐severe involvement. The identification of altered protein glycosylation is important for reaching a diagnosis. The clinical signs of PMM2 (phosphomannomutase‐2)‐CDG, the most common type of CDG, include psychomotor retardation with cerebellar hypoplasia, an abnormal fat distribution, inverted nipples, strabismus, hypotonia, failure to thrive, liver dysfunction, blood coagulation abnormalities, and cardiac and renal involvement. Some patients develop stroke‐like episodes (SLE) and severe systemic disorders that lead to early death. However, the phenotypes of CDG markedly vary. MPI (mannosephosphate isomerase)‐CDG is characterized by hypoglycemia, coagulation defects, and gastrointestinal and hepatic symptoms. Many other types of CDG are reported year by year, and CDG constitutes a significant area within inborn metabolic disorders [2].

Altered protein glycosylation is classified into *N*‐glycosylation defects, *O*‐glycosylation defects, and combined defects. The Type 1 pattern of *N*‐glycosylation disorders (CDG‐I) is a glycan assembly defect, while the Type 2 pattern (CDG‐II) is a glycan remodeling defect. The matrix‐assisted laser desorption/ionization (MALDI)–mass spectrometry (MS) analysis of tryptic peptides derived from transferrin is used to identify hypoglycosylated peptides characteristic of *N*‐glycosylation defects. Mass spectrometry was performed on apolipoprotein CIII (ApoCIII) to evaluate *O*‐glycan site occupancy and sialylation in CDG.

Glycan analyses of serum transferrin were initially performed via isoelectric focusing. Ohno et al. [3] and Wada et al. [4] reported CDG in Japan, which prompted a number of clinical studies on CDG in Japan [5, 6, 7, 8, 9, 10, 11]. A MS analysis revealed that the CDG‐I defect resulted from a metabolic error in the early glycosylation pathway [4].

Our research institute has contributed to the CDG diagnostic support center in Japan and developed diagnostic approaches. We have also participated in many recent studies on CDG in Japan [12, 13, 14, 15, 16, 17, 18, 19, 20, 21, 22, 23, 24]. The clinical findings are detailed in these references. Some other Japanese researchers reported rare types of CDG using WES [25, 26]. Advances in genetic analyses, including whole exome sequencing (WES), have revealed that other types of CDG are more common than previously considered. A comprehensive study on CDG in Japan has yet to be performed. Therefore, we herein investigated the clinical, biochemical, and genetic features of patients with CDG in our institution.

## 3 Materials and Methods

Patients with multisystem disease of unknown etiology in Japan were included in this study. The main clinical manifestations were mild‐to‐severe psychomotor disability, hypotonia, epileptic seizures, failure to thrive, dysmorphic features, endocrinological abnormalities, elevated transaminases, coagulopathy, and cerebellar hypoplasia. Patients with gastrointestinal symptoms are also included. The subjects of the analysis were primarily children under 10 years of age, with a particular emphasis on infants. A small number of adults were also included.

Frozen plasma or serum samples were sent to our research facility from medical institutions across Japan. The isoforms of transferrin and ApoCIII were examined by electrospray ionization MS (ESI‐MS) or MALDI‐MS. Wada reviewed the analytical methods for *N*‐ and *O*‐glycosylation disorders with MS [27]. The details of the analysis methods are provided in the Supporting Information. GPI‐anchor deficiencies, dystroglycanopathies, and glycosaminoglycan abnormalities belong to the broader CDG group [2], but they were excluded from this study.

In the initial analysis, cases with glycosylation abnormalities were often reanalyzed after a period of time. A genetic diagnosis was reached after identifying the glycoform abnormality. We also analyzed samples from patients with known variants or variants of unknown significance in the CDG associated genes identified by WES to confirm their pathogenicity. Variants identified in the Initiative on Rare and Undiagnosed Diseases (IRUD), a nationwide project, are a major source of pathogenic variants [28]. Informed consent was obtained from all subjects involved in the present study. Data, including clinical manifestations and complementary studies, were collected by the referring doctors. As this is a screening process, the possibility of false negatives is noted in the result report. If CDG cannot be ruled out, exome analysis and other molecular tests are recommended to the clinicians.

In addition, we conducted a literature review of reports on domestic cases of this disease.

## 4 Results

**FIGURE 1:**
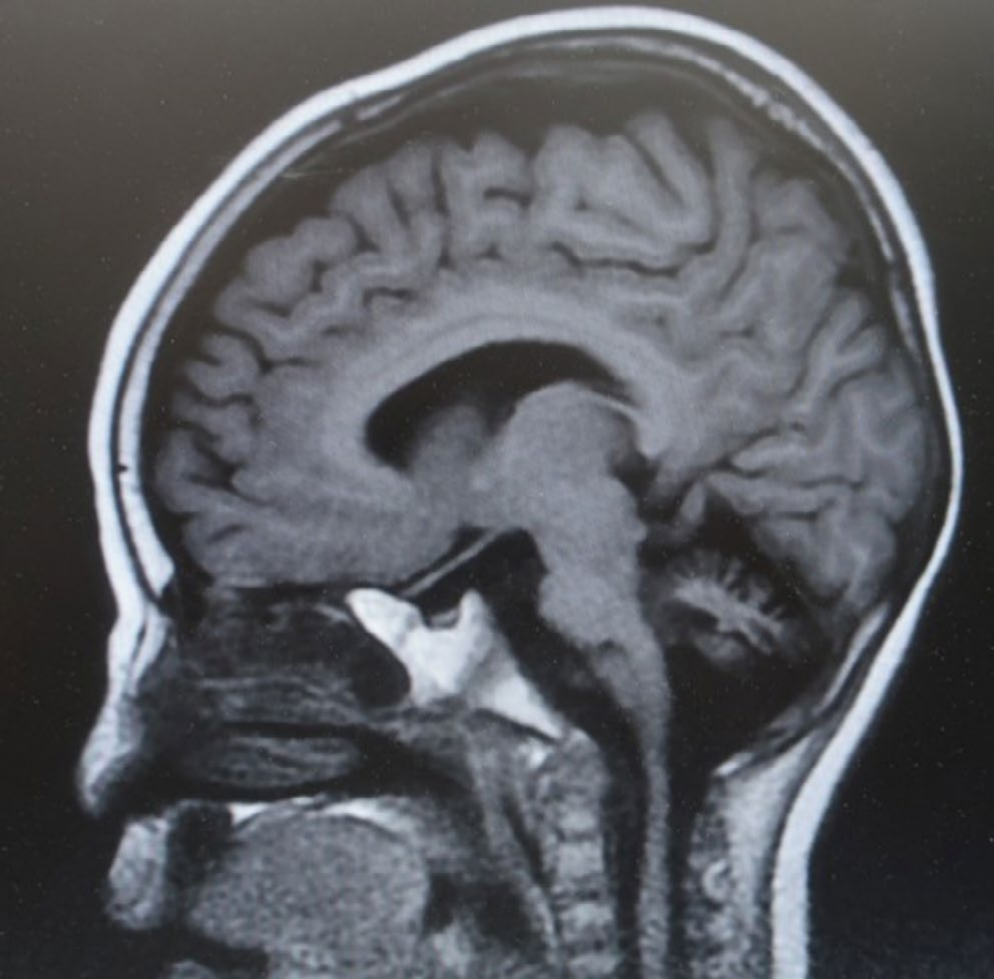
MRI image of PMM2‐CDG. This is a sagittal T1‐weighted MRI of the head of a 10‐year‐old boy with PMM2‐CDG, showing cerebellar atrophy. He suffered recurrent strokes, leading to progressive cerebellar atrophy. Additionally, developmental regression was observed in both cognitive and motor functions.

**FIGURE 2:**
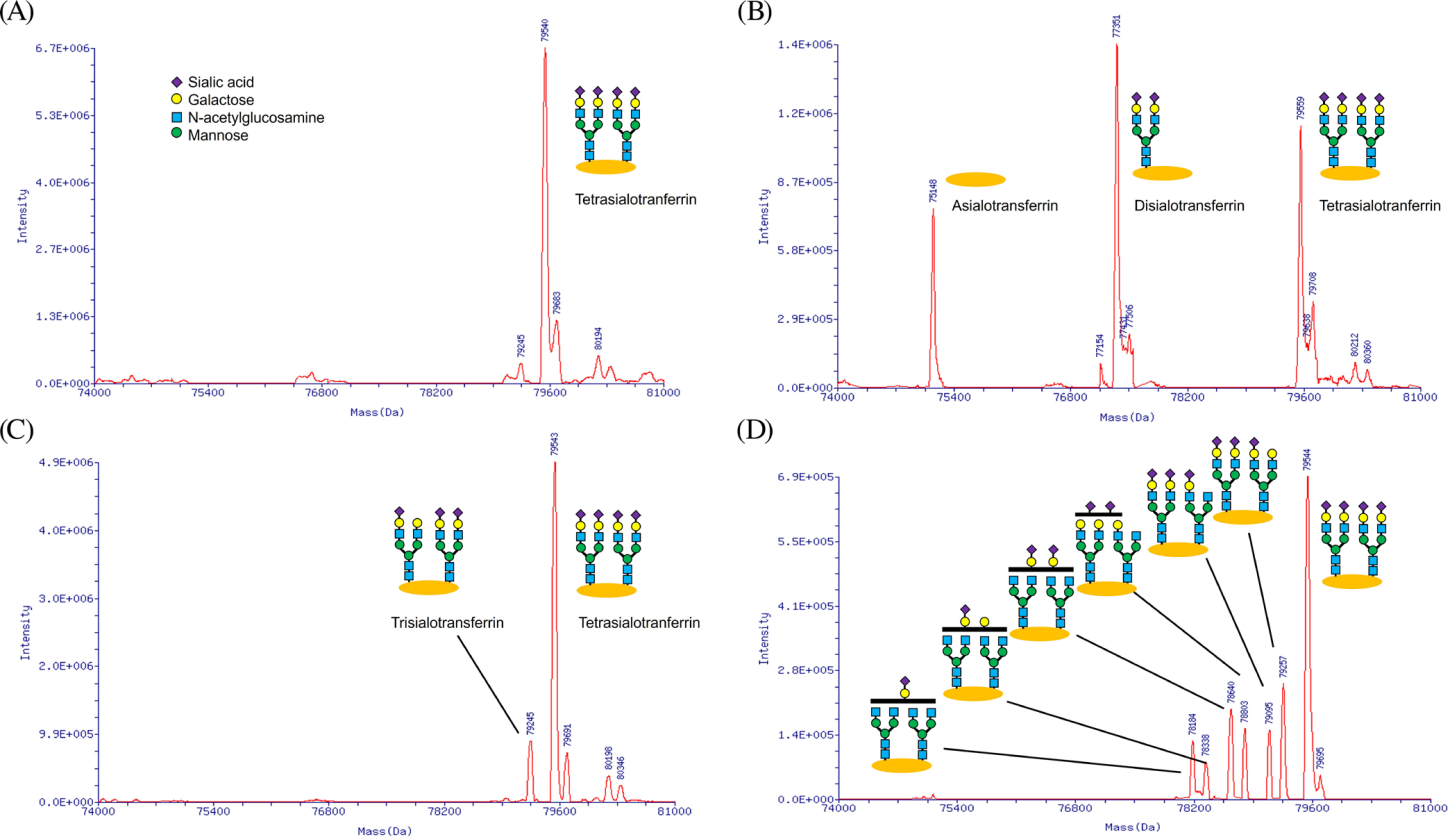
Electrospray ionization with quadrupole mass spectrometry of transferrin (deconvoluted spectrum). (A) Under normal conditions, tetrasialotransferrin accounts for the majority. (B) In PMM2‐CDG, disialotransferrin and asialotransferrin are increased. This figure shows the Type 1 pattern of *N*‐glycosylation defect. This is the analysis of a specimen from a 1‐year‐old male diagnosed with PMM2‐CDG. He presented with characteristic findings such as psychomotor developmental delay, hypotonia, elevated transaminases, and cerebellar atrophy. (C) In Type 2 CDG, trisialotransferrin is increased. Terminal sialic acid is lacking. (D) This is ATP6AP1‐CDG. Six patterns of abnormal glycans have been clearly identified by mass spectrometry. This infant male presented with liver dysfunction and coagulation abnormalities.

**FIGURE 3:**
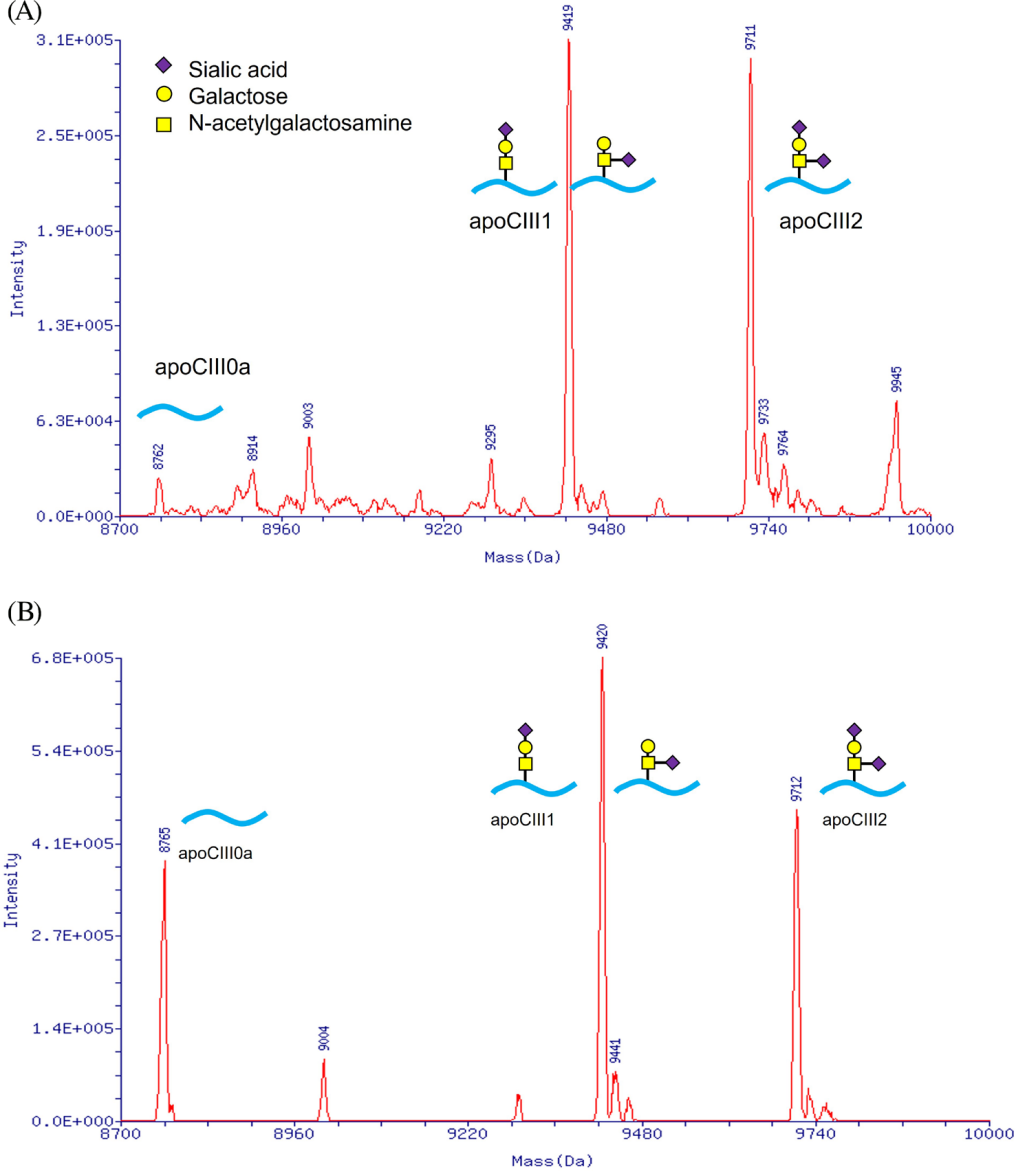
Electrospray ionization with quadrupole mass spectrometry of apolipoprotein C‐III (deconvoluted spectrum). (A) ApoCIII is a mucin‐type *O*‐glycosylation marker of CDG. ApoCIII0a (unglycosylated molecule), apoCIII1, and apoCIII2 are key diagnostic molecules whose relative abundances are important diagnostic parameters. The composition of ApoCIII0a, apoCIII1, and apoCIII2 in this figure is within the normal range. (B) This is a case of *O*‐glycosylation defect. ApoCIII0a is increased.

The phenotypic and genotypic spectra of 66 CDG patients during a long‐term (approximately 10 years) analytical study in one center are described herein. The patients were diagnosed with 23 different CDG types, including PMM2‐CDG, ALG1‐CDG, ALG6‐CDG, ALG9‐CDG, ALG12‐CDG, ALG14‐CDG, ATP6AP1‐CDG, ATP6AP2‐CDG, ATP6V0A2‐CDG, DHDDS‐CDG, GMPPA‐CDG, MAN1B1‐CDG, MOGS‐CDG, NANS‐CDG, NGLY1‐CDG, NUS1‐CDG, PGM1‐CDG, RFT1‐CDG, SLC35A1‐CDG, SLC35A2‐CDG, SLC39A8‐CDG, SRD5A3‐CDG, and SSR4‐CDG. A summary of CDG glycan analysis conducted at our institution is shown in Table 1. The most common was PMM2‐CDG, but it accounted for 17% of the total. Figure 1 shows an MRI of a 10‐year‐old boy with recurrent SLE, demonstrating cerebellar atrophy as a typical case of PMM2‐CDG. Representative glycan abnormalities detected by ESI‐MS are shown in the figures (Figures 2 and 3). Glycan analysis in urine was performed for some patients. All of the CDG types showed pathogenic or likely pathogenic variants confirmed by WES or Sanger sequencing. The details of the variants have been reported elsewhere or are planned to be reported, and therefore are not included in this paper.

**TABLE 1:** A summary of CDG glycan analysis conducted at our institution.

Genes	Number of patients	Type of CDG	References
*PMM2*	11	Type 1	[16, 19]
*ATP6V0A2*	6	Complex type	
*SLC35A2*	5	Type 2 or nomal sugar chain	[13, 14, 15]
*ALG9*	4	Type 2	
*SSR4*	4	Type 2	
*NANS*	3	Urinary excretion of ManNAc	[23]
*ALG6*	3	Type 1	[12]
*ALG14*	3	Type 1	
*ALG12*	2	Type 1	[17]
*MAN1B1*	2	Type 2	[21]
*MOGS*	2	Urinary excretion of Glc3Man	[18]
*PGM1*	2	Complex type	
*ATP6AP2*	2	Type 2, *O*‐linked	
*DHDDS*	2	Normal sugar chain	
*NUS1*	7 (2 families)	Normal sugar chain	[20]
*ATP6AP1*	1	Type 2	
*GMPPA*	1	Normal sugar chain	
*RFT1*	1	Type 1	
*ALG1*	1	Type 1	
*SLC39A8*	1	Type 2	
*SLC35A1*	1	Type 2	
*SRD5A3*	1	Type 1	[22]
*NGLY1* (CDDG)	1	Urinary excretion of *N*‐acetylglucosamine‐asparagine	[24]
Total	66		

*Note:* Type 1, Type 2: *N*‐glycosylation disorder. Complex type: *N*‐glycosylation disorder with *O*‐glycosylation disorder.

Some patients with apparent glycoform abnormalities did not show pathogenic variants in glycosylation‐related genes after WES. They were not included in the table. Further studies including whole genome analysis, mRNA analyses, and long‐read sequencing are planned for those patients.

## 5 Discussion

This is the first comprehensive study on CDG in Japan. The present study describes the clinical and molecular spectra of CDG in Japan. We reviewed reports on CDG in Japan in these 30 years. We have participated in the molecular diagnosis of 66 patients with CDG. The number of papers we have been involved in has increased significantly over the past decade. Frontiers in congenital disorders of glycosylation (FCDGC) Natural History cohort primarily based in Europe and the United States has registered 280 cases of CDG over a 5‐year period [29]. However, this number includes cases of GPI‐anchor deficiencies as well. It is presumed that a certain number of cases of CDG also exist in Japan.

Screening for CDG was performed by serum transferrin isoelectric focusing in the initial stage. Wada et al. [4] examined whole transferrin molecules using ESI‐MS and identified an abnormal species that was smaller than normal tetrasialotransferrin by 2200 Da, which is the size of the disialylated biantennary sugar chain. MS of whole transferrin molecules revealed glycosylation defects that were characteristic of the classical CDG‐I type of molecular abnormality [4]. Since the initial study, MS has played a key role in the identification and characterization of glycosylation defects affecting glycoproteins. MALDI‐MS of tryptic peptides derived from transferrin has been used to identify hypoglycosylated peptides that are characteristic of CDG‐I and II [27]. MS of glycopeptides from the tryptic digestion of transferrin delineates site‐specific glycoforms and has revealed the delicate balance of donor/acceptor substrates and the conformational effects of nascent proteins in cells [27]. ESI‐MS of transferrin with quadrupole mass analyzers for CDG was developed. In 2012, MALDI‐MS enabled rapid analyses without the prior separation of ApoCIII from serum [27]. MALDI‐MS of ApoCIII is a reliable method for elucidating the profiles of mucin‐type core *O*‐glycans, including site occupancy and glycoforms [27]. Wada et al. [30] performed ESI‐MS on ApoCIII to evaluate *O*‐glycan site occupancy and sialylation in CDG. These applications enabled the clarification of *N*‐glycosylation defects, *O*‐glycosylation defects, and combined defects.

If an abnormality in glycosylation is detected through MS, there is a possibility of false positives, meaning that this alone is insufficient for a definitive diagnosis. Genetic analysis is required for confirmation and subtype classification. In the case of the Type 1 pattern of *N*‐glycosylation disorders, genetic testing for PMM2‐CDG is typically performed. As discussed below, since PMM2‐CDG is rare in Japan, a genetic panel analysis or WES is usually conducted. Conversely, identifying genetic variants through exome sequencing alone does not confirm their pathogenic significance with certainty. Therefore, verifying glycosylation abnormalities using mass spectrometry is crucial. At our institution, such cases have been increasingly observed.

PMM2‐CDG is an autosomal recessive disorder that affects more than 1000 patients worldwide. Its estimated incidence is 1:20 000 in European countries. It is one of the most common inborn errors of metabolism in European countries. International clinical guidelines have been published for the management of PMM2‐CDG [31]. Muthusamy et al. [32] also indicated the direction for addressing the neurological issues of PMM2‐CDG. In PMM2‐CDG, cardiac involvement is a significant complication and can sometimes be a determining factor in prognosis [33]. In PMM2‐CDG, abnormalities in the hemostatic and coagulation system are closely related to acute events such as cerebral infarction and intracerebral hemorrhage [34].

Although the predominance of PMM2‐CDG in Japan was confirmed in our study, it only affects 17% of CDG. In FCDGC, excluding GPI‐anchor deficiencies, 94 out of 237 cases of CDG (40%) were classified as PMM2‐CDG. It is known that the frequency of p.Arg141His variant (dbSNP: rs28936415) is high in European countries. Pérez‐Cerdá et al. [35] reported the clinical, biochemical, and genetic features of patients with CDG in Spain in the last 20 years. Ninety‐seven patients were diagnosed with 18 different CDG. A total of 75% of patients had PMM2‐CDG presenting with a heterogeneous mutational spectrum including the variant of p.Arg141His (allele frequency of 22%). Asteggiano et al. [36] reported the 10‐year findings of screening for CDG in Argentina. They identified four individuals with PMM2‐CDG who carried the ancestral European disease causing the variant of p.Arg141His. Quelhas et al. [37] described the features of CDG diagnosed in Portugal in the last 20 years. Sixty‐three individuals were diagnosed with 14 distinct CDGs; serum transferrin isoelectric focusing revealed that 43 had the Type 1 pattern, 14 had the Type 2 pattern, and 2 had a normal pattern. The majority of patients (62%) had PMM2‐CDG. Lipinski et al. [38] reported 39 patients (from 35 families) with molecularly confirmed CDG in Poland, including 17 (44%) (from 16 families) with PMM2‐CDG.

Three studies conducted a molecular analysis of PMM2‐CDG in Japan [7, 8, 9]. Kondo et al. [7] detected missense variants in Exon 5 (p.Phe144Leu) and Exon 8 (p.Tyr229Ser, p.Arg238Pro) of the PMM2 gene. Mizugishi et al. [8] and Ono et al. [9] described a patient with PMM2‐CDG with a missense variant in exon 4 (p.Pro113Leu) and a novel nonsense mutation in Exon 7 (p.Arg194Ter). Including the 11 patients studied in our institution, p.Arg141His has not yet been identified in Japanese so far. The allele frequency of p.Arg141His in Europe is 0.005089, whereas that in East Asia is 0.0000. The low incidence of PMM2‐CDG in Japan is attributed to the low number of carriers of pathogenic gene variants.

Advances in genetic analyses, including WES, have revealed that other CDG are more common than previously considered. The present study reports the diagnosis of non‐PMM2 CDG patients. The manifestations of non‐PMM2‐CDG may include various neurological manifestations, such as hypotonia, intellectual disability, epilepsy, myopathy, myasthenia, movement disorders, cerebellar ataxia, and peripheral neuropathy. Extraneurological complications are cardiac abnormalities, gastrointestinal abnormalities, endocrine abnormalities, liver dysfunctions, cutaneous symptoms, immunodeficiency, coagulation defects, skeletal dysplasias, and ocular manifestations.

ATP6V0A2‐CDG (autosomal recessive cutis laxa, Type IIA) was detected in six unrelated patients. It is the second most common CDG in Japan. Hypotonia, dysmorphic features, cutis laxa, and neurological findings are the hallmarks of this syndrome. ATP6V0A2‐CDG shows combined *N*‐ and *O*‐glycosylation defects. Our applications enable the combined defects to be effectively clarified [39]. We performed ApoCIII *O*‐glycoform profiling on 500 serum samples by MALDI‐MS to diagnose CDG [40]. The content of unglycosylated ApoCIII was low in early infancy, indicating that *O*‐glycan occupancy needs to be assessed using age‐matched reference values. ATP6V0A2‐CDG is clinically easy to suspect, making it possible to perform genetic diagnosis by Sanger sequencing after confirming glycan abnormalities.

SLC35A2‐CDG is associated with early‐onset epileptic encephalopathies (EOEEs). Ng et al. [41] and Kodera et al. [13] described patients with pathogenic variants of *SLC35A2*. Further patients with SLC35A2‐CDG have been reported in Japan [14, 15]. SLC35A2‐CDG is an important disorder in the management of epilepsy. A recent report indicated the benefits of oral D‐galactose supplementation for SLC35A2‐CDG [42]. Other types of CDG often cause early‐onset epilepsy [43, 44]. It is important to note that EOEE and intractable seizures with unknown etiologies may be associated with CDG. Therefore, we suggest that all patients with EOEE be examined for CDG using a combination of MS and WES. Early treatment with D‐galactose supplementation may change the natural course of SLC35A2‐CDG.

However, analyses of serum proteins are not diagnostic in some cases of CDG. A serum transferrin analysis is not diagnostic in some cases of CDG. For example, the urinary excretion of Hex4 corresponding to Glc3Man is the biochemical hallmark of MOGS‐CDG [45], which was confirmed in a Japanese patient [19]. NANS‐CDG is caused by biallelic pathogenic variants in *NANS*, encoding an essential enzyme in *de novo* sialic acid synthesis [46]. The high urinary excretion of *N*‐acetylmannosamine (ManNAc) is pathognomonic in NANS‐CDG. Masunaga et al. [23] reported three patients with NANS‐CDG. Although the *N*‐glycosylation status of transferrin and the *O*‐glycosylation status of ApoCIII in serum were normal in these patients, the high urinary excretion of ManNAc was confirmed. We devised a measurement method by referring to the methodology of the paper by van Karnebeek et al. [46].

NGLY1‐associated congenital disorder of deglycosylation (CDDG1) is a rare autosomal recessive disorder caused by a functional impairment of the endoplasmic reticulum in the degradation of glycoproteins [47]. NGLY1‐CDDG is characterized by global developmental delay, microcephaly, epileptic seizures, hypotonia, involuntary movements, and alacrima or poor tear production. High urinary excretion of *N*‐acetylglucosamine‐asparagine (GlcNAc‐Asn) level was confirmed in a Japanese patient with NGLY1‐CDDG [23]. We devised a measurement method by referring to the methodology of the paper by Mueller et al. [48].

The different defects of CDG identified in Japan have expanded knowledge of CDG worldwide. Due to the low incidence of PMM2‐CDG, Japanese clinicians are not familiar with CDG; however, the present results confirm that certain numbers of patients with CDG are present in Japan. Quelhas and Jaeken [49] described a comprehensive overview of past and present approaches to the therapy of CDG. Various new treatment methods are being researched for CDG. Budhraja et al. [50] showed that liposome‐encapsulated mannose‐1‐phosphate therapy improves global *N*‐glycosylation in skin fibroblasts from individuals with PMM2‐CDG and some other CDG with enzymatic defects in early steps in protein *N*‐glycosylation. Shirakusa et al. [51] are advancing research toward the clinical application of this drug. There are reports of therapeutic research on other types of CDG, highlighting the need for accurate diagnosis.

The Japan Agency for Medical Research and Development launched the IRUD project in 2015 [28]. IRUD is an ambitious project that aims to construct a comprehensive medical network and internationally compatible data‐sharing framework. More than 7000 patients with undiagnosed diseases have been analyzed with trio‐based WES, and some were found to have variants associated with CDG. In neurological disorders, screening for inborn metabolic disorders is recommended before WES. Our institution received samples for CDG analysis associated with the IRUD project.

Wada [27] recently reported that the combination of the dried blood spot (DBS) method and triple quadrupole MS used for the newborn screening of inborn errors of metabolism was sufficient to characterize the aberrant glycoprofiles of Tf and ApoCIII in CDG. DBS or dried serum spot on filter paper can reduce the cost of sample transportation and induce mass spectrometric screening of CDG if effective treatment is developed.

## 6 Conclusion

This is the first comprehensive report on the clinical and molecular spectrum of CDG in Japan. Our data demonstrate that various types of CDG are present in Japan, with PMM2‐CDG being less common compared to European countries. Currently, our medical center is the only institution routinely performing molecular analysis of CDG. We suspect that undiagnosed CDG patients may exist. Therefore, expanding CDG analysis to more laboratories is essential in the future. Establishing a national system for the proper screening and management of CDG is a critical issue that needs to be addressed.

## Author Contributions

N.O. was responsible for the writing and editing of the final version of the manuscript. Y.W. contributed to the development of glycan analysis techniques. M.K. performed the practical tasks of mass spectrometry. All authors contributed to the study conceptualization, design, data collection, analysis, and interpretation of the data, as well as the drafting and revising of the manuscript.

## Ethics Statement

This study was approved by the institutional review board. This article does not contain any studies with animal subjects performed by any of the authors.

## Consent

The procedures in this study were performed in accordance with the ethical standards for medical research outlined in the Helsinki Declaration. Additional informed consent was obtained from all patients and/or parents for whom identifying information is included in this article.

## Conflicts of Interest

The authors declare no conflicts of interest.

## Supporting information


**Data S1.** Supporting Information.

## Data Availability

The data that support the findings of this study are available from the corresponding author upon reasonable request.
