# Supplementary material for: Clinical and Molecular Features of Patients With Congenital Disorders of Glycosylation in Japan
Source: JIMD Rep. 2025 Apr 4;66(3):e70011. doi: 10.1002/jmd2.70011 (PMC11969085; doi:10.1002/jmd2.70011)
Supplement: Supplementary file 1 — Data S1. Supporting Information. [file JMD2-66-e70011-s001.docx]

1. Purification of transferrin and apolipoprotein CIII (apoCIII)

Immunoaffinity columns were prepared using the corresponding antibodies, which are a rabbit (DAKO, Glostrup, Denmark) or a goat (Invitrogen, Thermo-Fisher Scientific, Waltham, MA) polyclonal antibody against human transferrin and a goat polyclonal antibody against human apoCIII (Academy Bio-Medical Co., Houston, TX, USA), and a ligand-coupling Sepharose column (HiTrap NHS-activated HP, GE Healthcare, Piscataway, NJ). Subsequently, the antibody-coupled Sepharose was recovered from the column. Ten μL of serum were mixed with a 20-μL slurry of the antibody-coupled Sepharose in 0.5 mL of phosphate-buffered saline (PBS), and the solution was incubated at 4°C for 30 min. After washing in PBS, the transferrin and apoCIII was eluted from Sepharose in 0.1 M glycine–HCl buffer at pH 2.5.

1. Electrospray ionization mass spectrometry (MS)

Liquid chromatography MS was carried out by an API4500 ESI-triple Q mass spectrometer (Sciex, Framingham, MA) connected to a C4 or C8 reversed phase desalting column (2 mm diameter and 10 mm length, GL Sciences, Tokyo) for transferrin and apoCIII, respectively.  After injection, the column was washed with 0.1 % formic acid at a flow rate of 0.2 mL/min, and then eluted with 60% acetonitrile/0.1 % formic acid at a flow rate of 0.05 ml/min.

For MS of transferrin, API4500 was operated in positive Q1 MS mode with the optimized parameters as follows: gas temperature was at 150 ℃, curtain gas pressure was 10 psi, ion source gas pressure was 16 psi, IonSpray voltage was 5.5 kV, declustering potential was 150 V, and entrance potential was 10 V. The full scan range was set from 1780 to 2000, and scan rate was 10 Da/s. For apoCIII, API4500 mass spectrometer was operated in positive Q1 MS mode with the parameters as follows: gas temperature was at 120 ℃, curtain gas pressure was 10 psi, ion source gas pressure was 16 psi, IonSpray voltage was 5.5 kV, declustering, or orifice-skimmer, potential (DP) was 100 V (50-150 V), and entrance potential was 10 V. The full scan range was set from 790 to 1650, and scan rate was 200 Da/s. Tandem mass spectrometry of m/z1388.3 ions was performed with collision energies of 50 V.

The zero-charge mass spectrum was generated by the Promass protein deconvolution software (Thermo-Fisher Scientific, Waltham, MA, USA).
